# Erratum

**DOI:** 10.1055/s-0041-1736228

**Published:** 2021-10-13

São Paulo, September 20, 2021

Dear readers,

In the *article Evaluation of Circular Saw Injuries in a Reference Center in Microsurgery and Reimplantation* ; DOI: 10.1055/s-0041-1729940 .), published online in Rev Bras Ortop in August 27, 2021

**Where it reads (English version):**

Kátia Campos dos Anjos

Department of Social Health, Instituto de Ortopedia e Traumatologia, Hospital das Clínicas, Faculdade de Medicina, Universidade de São Paulo (IOT-HCFMUSP), São Paulo, SP, Brazil

**It should read:**

Kátia Campos dos Anjos

Social Service, Instituto de Ortopedia e Traumatologia, Hospital das Clínicas, Faculdade de Medicina, Universidade de São Paulo (IOT-HCFMUSP), São Paulo, SP, Brazil

**Where it reads (Portuguese version):**

Kátia Campos dos Anjos

Departamento de Assistência Social, Instituto de Ortopedia e Traumatologia, Hospital das Clínicas, Faculdade de Medicina, Universidade de São Paulo (IOT-HCFMUSP), São Paulo, SP, Brasil

**It should read:**

Kátia Campos dos Anjos

Serviço Social, Instituto de Ortopedia e Traumatologia, Hospital das Clínicas, Faculdade de Medicina, Universidade de São Paulo (IOT-HCFMUSP), São Paulo, SP, Brasil

**Also, the author Angela dos Santos should have been included as an author of this article:**

Angela dos Santos

Programa de Especialização em Serviço Social em Ortopedia, Instituto de Ortopedia e Traumatologia, Hospital das Clínicas, Faculdade de Medicina, Universidade de São Paulo (IOT-HCFMUSP), São Paulo, SP, Brasil

