# Supplementary material for: Erratum
Source: Rev Bras Ortop (Sao Paulo). 2021 Oct 13;59(6):e1012. doi: 10.1055/s-0041-1736228 (PMC11663070; doi:10.1055/s-0041-1736228)
Supplement: Related texts

# Correction: Errata

São Paulo, 20 de setembro de 2021

Prezados leitores,

No artigo “Avaliação de lesões por serra circular em um centro de referência em microcirurgia e reimplantes” (DOI: 10.1055/s-0041-1729940 ), publicado *on-line* na Rev Bras Ortop em 27 de agosto de 2021

Onde se lê (na versão em inglês):

Kátia Campos dos Anjos

Department of Social Health, Instituto de Ortopedia e Traumatologia, Hospital das Clínicas, Faculdade de Medicina, Universidade de São Paulo (IOT-HCFMUSP), São Paulo, SP, Brazil

**Deve-se ler:**

Kátia Campos dos Anjos

Social Service, Instituto de Ortopedia e Traumatologia, Hospital das Clínicas, Faculdade de Medicina, Universidade de São Paulo (IOT-HCFMUSP), São Paulo, SP, Brazil

**Onde se lê (versão em português):**

Kátia Campos dos Anjos

Departamento de Assistência Social, Instituto de Ortopedia e Traumatologia, Hospital das Clínicas, Faculdade de Medicina, Universidade de São Paulo (IOT-HCFMUSP), São Paulo, SP, Brasil

**Deve-se ler:**

Kátia Campos dos Anjos

Serviço Social, Instituto de Ortopedia e Traumatologia, Hospital das Clínicas, Faculdade de Medicina, Universidade de São Paulo (IOT-HCFMUSP), São Paulo, SP, Brasil

**Além disso, a autora Angela dos Santos deve ser incluída como autora deste artigo:**

Angela dos Santos

Programa de Especialização em Serviço Social em Ortopedia, Instituto de Ortopedia e Traumatologia, Hospital das Clínicas, Faculdade de Medicina, Universidade de São Paulo (IOT-HCFMUSP), São Paulo, SP, Brasil.
